# Future Directions of Robotic Surgery: A Case Study of the Cornell Athermal Robotic Technique of Prostatectomy

**DOI:** 10.1100/tsw.2006.395

**Published:** 2006-03-19

**Authors:** Robert A. Leung, Tara S. Kim, Ashutosh K. Tewari

**Affiliations:** Weill Medical College of Cornell University, Department of Urology, New York, USA

**Keywords:** prostate neoplasms, robotics, prostate imaging, instrumentation, surgery

## Abstract

Robotic radical prostatectomy (RRP) has become an effective modality in the treatment of localized prostate cancer. We detail the experience at our institution and provide a perspective for future considerations of RRP with respect to improved preoperative imaging and surgical instrumentation.

## INTRODUCTION

Robotics is here. A Medline search for the term “robotic surgery” yielded 191 matches published in 2005. Robotic surgery is applicable to a wide array of urologic procedures. The benefits include improved vision through magnified stereoscopic magnification and enhanced movement through tremor filtration and an expanded range of motion.

While these enhanced tools help to simplify complex surgical tasks, there is a need to develop technology further to tailor surgical care for men with prostate cancer. We believe that the future of robotics will be within the realm of improved imaging and instrumentation. The purpose of this review is to provide a perspective on the current state and future directions of robotic radical prostatectomy with respect to these domains.

## BACKGROUND

Prostate cancer is the most common nonskin malignancy in men in the U.S. It is estimated that in 2006, 234,460 new incident cases will emerge and 27,350 men will die from the disease [1]. Over the past decade, with the widespread use of prostate-specific antigen (PSA) screening, stage migration has occurred. More men are presenting with organ-confined disease and have a longer estimated life expectancy at the time of diagnosis[2,3]. There remains considerable controversy about the ideal management of the disease.

Radical prostatectomy is the most commonly recommended treatment for patients with a life expectancy greater than 10 years[4]. Radical prostatectomy has been shown to reduce disease-specific mortality by nearly 50% as compared to expectant management in a recent randomized trial[5]. Erectile dysfunction and urinary incontinence are the most common morbidities associated with radical prostatectomy. These morbidities have been decreased since the anatomic foundations of the nerve-sparing radical retropubic prostatectomy (RRP) approach were described by Walsh et al.[6,7].

Robotic radical prostatectomy (RRP) was developed in order to further reduce perioperative morbidity. In the U.S., RRP was developed at the Henry Ford Hospital (Detroit, MI), using the da Vinci surgical system (Intuitive Surgical, Sunnyvale, CA)[8,9]. Robotic prostatectomy conceptually mimics the gold-standard open radical retropubic prostatectomy approach, with decreased surgical invasiveness. The current treatment paradigm for the potent prostate cancer patient can be distilled into the following triad, 1) the elimination of the cancer while 2) preserving the anatomic structures necessary for urinary continence and 3) preserving the neural structures necessary for sexual potency. Preliminary data on robotics have shown acceptable rates in effectively accomplishing these goals, however, future directions in robotics will serve to enhance the treatment of patients.

## THE ROLE OF ROBOTICS IN CANCER CONTROL, NERVE SPARING, AND CONTINENCE PRESERVATION

Foundations for the surgical technique of RRP has been described elsewhere[10,11,12,33,34,35,36,37]. The technique at our institution is a modified version of the Vattikuti Institute Prostatectomy (VIP) procedure. Below, we describe our technique and report the results of a consecutive series of surgeries performed at our institution[13,14].

During the period between January to December 2005, 200 RRP and bilateral pelvic lymphadenectomies were performed at our institution by a single surgeon (AKT). We reviewed the clinicopathologic and operative data for these patients through an institutional review board-approved prospective database protocol. Patients with localized prostate cancer who are candidates for open radical prostatectomy were offered robotic prostatectomy. We did not exclude any patient on the basis of surgical consideration alone, including elevated body mass index, previous abdominal or genitourinary surgery, or prostate volume.

### Utility of Robotic Technology in Controlling Margin Status

The benefits of the robotic instrument have been documented elsewhere[37]. Briefly, robotic visualization and versatility of the instrument help in finding a right plane outside the prostatic capsule. Vision also helps to recognize variations in tissue texture, color, obliteration of planes, adhesions, tissue recognition, and anatomic planes. A relatively bloodless field and three-dimensional depth perception helps when making critical decisions about the plane of dissection when there is a suspicion of extracapsular extension or capsular incision. The ability to control miniaturized scissors and forceps through narrow planes behind and around the prostate, make awkward bends at the wrist of the instrument and precisely dissect the nerves all contribute significantly to the goal of achieving a negative margin. As reported below, our pathologic data is quite acceptable even in T3 cancers. As will be seen in next paragraph, our pathologic data are quite acceptable even in T3 cancers.

Pathologic data are presented in Table 1. Clinically localized neoplasms made up 90% of our cohort (n = 181). Our overall margin rate is 6%. The positive surgical margin (SM+) rate (pT2/SM+) within organ-confined malignancies was 4.9%. Furthermore, the SM+ rate in pT3 cancers was 11.7%. The SM+ rate for the past 100 cases is 3.0%, with a SM+/pT2 (2/90) = 2.2%.

Likewise, in previously published data on RRP, pathological outcomes compare favorably to open radical prostatectomy. The SM+ rate for organ-confined cancers for published RRP series greater than 40 cases are between 4.5 and 14.8%[15,16]; a recent report on a large cohort of RRP reported a SM+ rate of 7.7%[17]. Therefore, it appears that in expert hands, cancer control using robotic assistance promises as good of an outcome as the gold-standard open radical prostatectomy.

**TABLE 1**
**Pathological Margin Data for 200 Consecutive Robotic Cases**

| | SM– | SM+ | Total |
|---|---|---|---|
| T0 | 1 | 0 | 1 |
| T2a | 28 | 0 | 28 |
| T2b | 10 | 0 | 10 |
| T2c | 133 | 9 | 142 |
| T3a | 10 | 2 | 12 |
| T3b | 5 | 0 | 5 |
| T3c | 1 | 0 | 1 |
| T4a | 0 | 1 | 1 |
| Total | 188 | 12 | 200 |
| Overall SM+ | 6% | | |
| Organ-Confined SM+ | 4.9% | | |
| Last 100 Cases | SM+/pT2 = 2/90 = 2.2% | SM+/pT3 = 1/10 = 10% | |

## Utility of Robotic Instruments in Preserving Continence

The use of microinstruments during radical prostatectomy is of great value for the surgeon in order to preserve structures related to urinary continence. The puboprostatic ligaments, puboperinealis muscles, and arcus tendineous (condensation of the endopelvic fascia) are all recognized as important supporting structures for urinary continence in both men and women. Using robotic instruments, we performed cadaveric studies to devise innovative strategies to achieve early continence. We preserved these structures and recreated an intact puboprostatic and arcus tendineous complex at the end of RRP. We developed a plan to reconnect the ligaments to the urethrovesical anastomosis, reapproximated the muscle, and fixed the distal bladder to the arcus tendineous. These modifications were attempted in a series of 25 consecutive patients undergoing RRP at our institution. Patients were asked to report their continence outcomes. We compared their results to a consecutive series of 25 previous patients who did not receive this modification[38].

The technique was reproducible in all 25 patients and without substantial addition to operative time (t = 5 min). At 4 weeks, 65% of these patients were continent (0–1 pad daily) vs. our control group with a continence rate of 46%. There were no differences in surgical margin rates for these patients. We suspect that the continence rates for both cohorts will converge eventually, although the preservation of the puboprostatic ligaments, puboperinealis muscles, and arcus tendineous may help in an earlier return to continence.

## Technical Innovations in Preservation of Nerves During Robotic Prostatectomy

Although the roadmap for nerve sparing during radical retropubic prostatectomy was laid down by Walsh et al.[6,7], these anatomic principles need to be re-emphasized in the context of robotic surgery. This is because surgical steps are reversed, visual angles are different, and magnification and stereoscopy provide more detailed anatomical images than that seen by surgeons during open surgery. We performed anatomic studies involving 10 fresh male cadavers and collected video and still image data from 200 patients undergoing radical prostatectomy using the Athermal Robotic Technique at Weill Medical College of Cornell University, NY. We report on the experience below.

From a surgical standpoint, a trizonal neural architecture was noted. This included the proximal neurovascular plate (PNP), the predominant neurovascular bundle (PNB), and accessory neural pathways (ANPs). The PNP was located 5–10 mm (average 5 mm) lateral to the seminal vesicles, was 2–7 mm (average 3 mm) thick, 5–25 mm (average 7 mm) wide, and 4–30 mm in length (average 9 mm). It was located within 4–15 mm (average 6 mm) of the bladder neck, 2–7 mm (average 5 mm) of endopelvic fascia, and overlapped 0–7 mm (average 5 mm) of the proximal prostate. The PNB varied in its shape and size from the proximal to distal end, was thickest at the base, and was most variable in course and architecture near the apex. In 65% of cases, a medial extension was noted behind the prostate, which converged medially at the apex in 30% of cases. There were significant variations in the architecture and thickness of the bundle. ANPs were noted within the layers of levator fascia on the anterolateral aspect in 40% of cases and on the posterior aspect of the prostate in 25% of cases. In 75% of the cadavers, the proximal third of the prostate was covered by the PNP where these accessory nerve fibers were most prominent. These accessory branches formed a plexus on the posterolateral aspect of the apex in 35% of cases[39].

Based on these findings and modifications to our surgical approach, including preservation of all three levels of nerves and avoiding the use of cautery during nerve sparing, we have been able to achieve 45% return of sexual function at 6 weeks in patients undergoing bilateral nerve sparing.

## FUTURE INNOVATIONS

Robotic technology renders itself to improved visualization and decision-making capabilities by incorporating data from multiple imaging sources. These can include large three-dimensional displays and real-time tissue visualization using confocal microscopy, second generation harmonic laser visualization, and neural mapping. Robotics is also ready to integrate with nanotechnology and possibly serve as a delivery tool for targeted therapeutics and miniaturized surgical tools. Presented herein is the summary of such innovations.

### Magnetic Resonance Imaging (MRI) and Proton Magnetic Resonance Spectroscopy (MRSI)

Two of the most promising tools for noninvasive, anatomic, and metabolic evaluation of prostate cancer are magnetic resonance imaging (MRI) and proton magnetic resonance spectroscopy (MSRI)[20]. Many times, the decision to preserve or resect the neurovascular bundle is based on incomplete information about tumor aggressiveness. We hypothesize that this shortcoming of preoperative tumor assessment plays a significant role in cancer control and margin positivity. For example, gleason grade is a good predictor of aggressiveness, although there is weak correlation between transrectal ultrasound (TRUS) guided biopsy grade and final pathology grade [21]. Also, regions not palpable on digital rectal examination (DRE), such as the anterior peripheral, transitional, or central zones, pose significant problems when assessing specific tumor location[22]. These problems are circumvented by MSRI.

MSRI provides a three-dimensional metabolic map of the prostate gland. Normal prostatic tissue contains high levels of citrate. Prostate lesions demonstrate increased levels of choline and often decreased levels of citrate. This phenomenon can be explained by a high phospholipids cell membrane turnover in the malignant tissue and a conversion from citrate-producing to citrate-oxidizing cancer metabolism. Tumor detection and gradation is based on an increased ratio of choline to citrate and a decreased or absent level of polyamines[23].

Several studies integrating MSRI into the current treatment matrix for prostate cancer including MRI, TRUS-guided biopsy, and DRE have yielded positive results. Also, MRI and MSRI used together have shown the ability to detect cancer in the peripheral zone[24]. The ability of MRI and MSRI to detect lesions in areas not normally biopsied may decrease the rate of false-positive biopsies by either guiding where biopsies should be performed or eliminating the need for repeat biopsy in patients with high serum PSA[25]. In addition, it has been shown that MSRI can effectively predict extracapsular extension (ECE)[26].

Current research is being performed to identify and better understand metabolic markers for prostate cancer. Hricak reports that at high field strengths, certain molecular markers can be detected on MSRI (e.g., cell proliferation markers and apoptosis markers). For example, the marker Ki-67 has shown utility in predicting tumor progression, treatment outcome, and biochemical recurrence[23].

In summary, imaging, with respect to MR and MSRI, has shown utility in detecting the specific locations of cancerous lesions within the prostate. The integration of these imaging techniques along with the improved precision offered with robotic prostatectomy, through a magnified three-dimensional view and tremor filter, will further assist in reducing SM+ rates.

## Improved Instrumentation and Augmented Reality

A common problem with the current robotic system is the lack of tactile feedback afforded to the surgeon[27]. This problem is being addressed by several laboratories. The Laboratorie de Robotique de Paris is developing a sensor that measures the forces and torques applied by the surgeon[28]. The sensor is located outside the body and is placed between the trocar and the handle of the instrument. The robotic arm drives the instrument and keeps exerted forces within a certain limit.

Furthermore, a series of specialized end-effectors are being developed for use with robotic surgical systems. The Endo Stitch™ (Tyco Healthcare) is a tool used to simplify the endoscopic suturing technique[29]. The Endo-Stitch™ utilizes a needle that is 9 mm long and 0.9 mm wide. The suture is attached to the middle of the needle. The advantage of the device is the lack of continuous grasping during suturing.

As can be seen with the Endo-Stitch™, we suspect that a niche device market, aimed at completing singular tasks (i.e., suturing), will emerge in order to take advantage of the improved dexterity of robotic systems. This may include probes to provide real-time nerve imaging[30] and mapping of the surgical area or augmented reality (AR).

Marmulla and associates have shown that computer-generated navigation maps can be projected right onto the surgical site during craniomaxillofacial surgery[31]. Structural data, through CT scans, are entered into an algorithm. These data are, in turn, projected onto the surgical surface through a high beam of light. Furthermore, Falk et al. have shown that such a system can be used during endoscopic coronary artery bypass grafting (CABG) using a robotic system[32]. The foundations set by other surgical specialties may help to develop such a system for use in urologic procedures.

## Real-Time Nerve Imaging with Second Harmonic Generation and Multiphoton Microscopy

Second harmonic generation (SHG) and multiphoton microscopy (MPM) provide the basis for quick and safe visualization of nervous tissue during RRP. We have performed the preliminary studies to develop such an endoscope for use during the procedure.

Multicolor, nonlinear microscopy of living tissue using two- and three-photon-excited intrinsic fluorescence combined with SHG by supermolecular structures produces images with the resolution and detail of standard histology without the use of exogenous stains. Imaging of intrinsic indicators within tissue, such as nicotinamide adenine dinucleotide, retinol, indoleamines, and collagen provides crucial information for physiology and pathology. Intrinsic emission of MPM enables three-dimensional imaging of unstained living tissue. MPM is proving useful as an effective alternative to standard histopathological preparations for rapid examination of fresh tissues[30]. In preliminary studies, we have been able to differentiate normal prostatic cells from nonprostatic tissue. Furthermore, SHG has been shown to be effective in delineating nerve axon bundles.

This technique can visualize the structure and function of nervous tissue under physiological and pathophysiological conditions. The advantages are that native, unstained tissue can be investigated "as is" and that the signal originates directly from the intracellular molecule of interest. This technique can provide subcellular images of tissue and can help to identify various tissues with greater precision. More directly, we conceive that the nerves could be differentiated from the prostate, sphincter, and prostatic apex.

## CONCLUSIONS

Robotic prostatectomy is here. It offers a minimally invasive, surgical approach for men with localized prostate cancer. Short-term clinical and pathological results compare favorably with open radical retropubic prostatectomy.

We believe that the operating room of the future will better meet the goals of (1) tumor identification, (2) identification of structures important to potency preservation, and (3) identification of structures vital to preservation of urinary continence. The next 5 years will be important to assess radical prostatectomy as a viable cancer-control option for localized prostate cancer. Datasets will mature and outcomes with respect to erectile dysfunction and urinary incontinence will become available. Advances in pretreatment imaging and improved instrumentation offer the promise of improved patient outcomes.

## References

1. Jemal, A., Siegel, R., Ward, E., et al. (2006) Cancer Statistics. *CA Cancer J. Clin.* **56,** 106-30.
2. Han, M., Partin, A.W., Chan, D.Y., et al. (2004) An evaluation of the decreasing incidence of positive surgical margins in a large retropubic prostatectomy series. *J. Urol.* **171,** 23–26.
3. Derweesh, I.H., Kupelian, P.A., Zippe, C., et al. (2004) Continuing trends in pathological stage migration in radical prostatectomy specimens. *Urol. Oncol.* **22,** 300–306.
4. World Health Organization, Stewart, B.W., and Kleihues, P., Ed. (2003) World Cancer Report. International Agency for Research on Cancer, Lyon.
5. Holmberg, L., Bill-Axelson, A., Helgesen, F., et al. (2002) A randomized trial comparing radical prostatectomy with watchful waiting in early prostate cancer. *N. Engl. J. Med.* **347**, 781–789.
6. Walsh, P.C. and Donker, P.J. (1982) Impotence following radical prostatectomy: insight into etiology and prevention. *J. Urol.* **128,** 492–497.
7. Walsh, P.C., Lepor, H., and Eggleston, J.C. (1983) Radical prostatectomy with preservation of sexual function: anatomical and pathological considerations. *Prostate* **4,** 473–485.
8. Menon, M., Tewari, A., Peabody, J.O., et al. (2004) Vattikuti Institute prostatectomy, a technique of robotic radical prostatectomy for management of localized carcinoma of the prostate: experience of over 1100 cases. *Urol. Clin. North Am.* **31,** 701–717.
9. Menon, M., Shrivastava, A., Tewari, A., et al. (2002) Laparoscopic and robot assisted radical prostatectomy: establishment of a structured program and preliminary analysis of outcomes. *J. Urol.* **168**, 945–949.
10. Tewari, A., Peabody, J., Sarle, R., et al. (2002) Technique of da Vinci robot-assited anatomic radical prostatectomy. *Urology* **60,** 569–572.
11. Menon, M., Tewari, A., Baize, B., et al. (2002) Prospective comparison of radical retropubic prostatectomy and robot-assisted anatomic prostatectomy: the Vattikuti Urology Institute experience. *Urology* **60**, 864–868.
12. Sarle, R., Tewari, A., Shrivastava, A., et al. (2004) Surgical robotics and laparoscopic training drills. *J. Endourol.* **18,** 63–66.
13. Tewari, A.K. (2005) Advanced Robotic Techniques (ART) Radical Prostatectomy. Surgical Telecast and Lecture. Presented at the 1st Meeting of Minimally Invasive Robotic Association (MISA), Innsbruck, Austria, December 8, 2005.
14. Tewari, A.K. (2005) State of ART (Advanced Robotic Technique). Lecture presented at New York Section AUA Meeting, Vienna, Austria, September 20, 2005.
15. Ahlering, T.E., Skarecky, D., Lee, D., et al. (2003) Successful transfer of open surgical skills to a laparoscopic environment using a robotic interface: initial experience with laparoscopic radical prostatectomy. *J. Urol.* **170,** 1738–1741.
16. Ahlering, T.E., Woo, D., Eichel, I., et al. (2004) Robot-assisted versus open radical prostatectomy: a comparison of one surgeon's outcomes. *Urology* **63,** 819–822.
17. Han, M., Partin, A.W., Chan, D.Y., et al. (2004) An evaluation of the decreasing incidence of positive surgical margins in a large retropubic prostatectomy series. *J. Urol.* **171,** 23–26.
18. Hull, G.W., Rabbani, F., Abbas, F., et al. (2002) Cancer control with radical prostatectomy alone in 1,000 consecutive patients. *J. Urol.* **167**, 528–534.
19. Roehl, K.H., Han, M., Ramos, C.G., et al. (2004) Cancer progression and survival rates following anatomical radical retropubic prostatectomy in 3,478 consecutive patients: long term results. *J. Urol.* **172,** 910–914.
20. Hricak, H. (2005) MR imaging and MR spectroscopic imaging in pre-treatment evaluation of prostate cancer. *Br. J. Radiol.* **78,** S103–S111.
21. Zakian, K.L., Sircar, K., Hricak, H., et al. (2005) Correlation of proton MR spectroscopic imaging with gleason score based on step-section pathologic analysis after radical prostatectomy. *Radiology* **234**, 804–814.
22. Wefer, A.E., Hricak, H., Vigneron, D.B., et al. (2000) Sextant localization of prostate cancer: comparison of sextant biopsy, magnetic resonance imaging and magnetic resonance spectroscopic imaging with step section histology. *J. Urol.* **164,** 400–404.
23. Hricak, H. New horizons in genitourinary oncologic imaging. *Abdominal Imaging.* Available at http://www.springerlink.com/media/84v16cpkgk1jnga07q96/contributions/7/3/1/6/73161r43688u4417_html/fulltext.html
24. Scheidler, J., Hricak, H., Vigneron, D.B., et al. (1999) Prostate cancer: localization with three-dimensional proton MR spectroscopic imaging—clinicopathologic study. *Radiology* **213**, 473–480.
25. Terris, M.K. (2002) Prostate biopsy strategies: past, present, and future. *Urol. Clin. North Am.* **29,** 205–212.
26. Wang, L., Mullerad, M., Chen, H.N., et al. (2004) Prostate cancer: incremental value of endorectal MR imaging findings for prediction of extracapsular extension. *Radiology* **232**, 133–139.
27. Cepolina, F., Challacombe, B., and Michelini, R.C. (2005) Trends in robotic surgery. *J. Endourol.* **19,** 940–950.
28. Zemiti, N., Ortmaier, T., Vitrani, M., et al. (2004) A force controlled laparoscopic surgical robot without distal force sensing. Present at ISER 2004: 9$^{th}$ International Symposium on Experiemental Robotics, Singapore, June 18–21, 2004.
29. Autosuture Division, Tyco Healthcare. Endo-Stitch™. http://www.autosuture.com
30. Zipfel, W.R., Williams, R.M., Christie, R., et al. (2003) Live tissue intrinsic emission miscroscopy using multiphoton-excited native fluorescence and second harmonic generation. *Proc. Natl. Acad. Sci. U. S. A.* **100,** 7075–7080.
31. Marmulla, R., Hoppe, N., Muhling, J., et al. (2005) An augmented reality system for image-guided surgery. *Int. J. Oral Maxillofac. Surg*. **34,** 594–596.
32. Falk, V., Mourgues, F., Adhami, L., et al. (2005) Cardio navigation: planning, simulation, and augmented reality in robotic assisted endoscopic bypass grafting. *Ann. Thorac. Surg*. **79**, 2040–2048.
33. Badani, K.K., Bhandari, A., Tewari, A., et al. (2005) Comparison of two-dimensional and three-dimensional suturing: is there a difference in a robotic setting? J*. Endourol.* **19**, 1212–1215.
34. Menon, M., Shrivastava, A., and Tewari, A. (2005) Laparoscopic radical prostatectomy: conventional and robotic. *Urology* **66(5 Suppl),** 101–104.
35. Hemal, A.K., Bahndari, A., Tewari, A., et al. (2005) The window sign: an aid in laparoscopic and robotic radical prostatectomy. *Int. Urol. Nephrol*. **37**, 73–77.
36. Tewari, A., El-Hakim, A., Horninger, W., et al. (2005) Nerve-sparing during robotic radical prostatectomy: use of computer modeling and anatomic data to establish critical steps and maneuvers. *Curr. Urol. Rep*. **6,** 126–128.
37. Tewari, A., Kaul, S., and Menon, M. (2005) Robotic radical prostatectomy: a minimally invasive therapy for prostate cancer. *Curr. Urol. Rep*. **6**, 45–48.
38. Tewari, A.K., Schiff, J., Bigelow, K., et al. (2006) Neuroanatomic Maps for Nerve Sparing Robotic Prostatectomy: A Study Describing the Importance of the Neurovascular Plate and Its Relationship to the Steps of Surgery. Abstract accepted for presentation at the AUA (American Urological Association) Annual Meeting, Atlanta, GA, May 20–25, 2006.
39. Takenaka, A., Tewari, A., Hara, R., et al. (2006) Pelvic autonomic nerve mapping around the prostate by intraoperative electrical stimulation with simultaneous measurement of intracavernous and intraurethral pressure. *J. Urol.*, submitted for publication.
